# Comparison of short‐ and long‐term objective respiratory outcomes after surgery for brachycephalic obstructive airway syndrome

**DOI:** 10.1111/vsu.70034

**Published:** 2025-10-18

**Authors:** Daisy A. Johnson, Nai‐Chieh Liu, Jane F. Ladlow

**Affiliations:** ^1^ Small Animal Teaching Hospital University of Liverpool Neston UK; ^2^ School of Veterinary Medicine, Institute of Veterinary Clinical Science National Taiwan University Taipei Taiwan; ^3^ Granta Veterinary Specialists Cambridge UK; ^4^ Department of Veterinary Medicine University of Cambridge Cambridge UK

## Abstract

**Objectives:**

To report and compare short‐ and long‐term outcomes in dogs following surgery for brachycephalic obstructive airway syndrome (BOAS).

**Study design:**

Longitudinal cohort study.

**Sample population:**

Client owned dogs (*n* = 32).

**Methods:**

Dogs that underwent BOAS surgery before 2019 with preoperative and short‐term postoperative assessments were recruited for long‐term follow up to obtain respiratory functional grades (RFG) and BOAS indices. Dogs that underwent a second airway surgery (33 of 117) were excluded. Comparisons of BOAS indices and RFGs among preoperative, short‐term, and long‐term postoperative time points were performed using Friedman's tests and post hoc Wilcoxon signed rank tests with Bonferroni corrections.

**Results:**

There were 32 of 117 dogs available for long‐term assessment. Median long‐term postoperative assessments occurred at 1645 days after surgery (range 1208–2927 days). Long‐term postoperative RFG and BOAS index values were improved compared with preoperative values. There was no difference between short‐term and long‐term postoperative assessments (*p* > .999 for RFG values, and *p* = .623 for BOAS index values). Owners reported a high degree of satisfaction with surgery: 55% believed their dogs no longer had breathing problems (long‐term BOAS index 50.8 ± 17.6%) and 39% believed their dog still had breathing problems (long‐term BOAS index 48.9 ± 20.5%).

**Conclusion:**

Improvements in RFG and BOAS Indices seen following surgery were maintained over time.

**Clinical significance:**

This is the first study with clinician‐assessed objective long‐term respiratory outcomes of BOAS surgery. The long‐term improvements in RFG and BOAS indices support the long‐term clinical benefit and durability of surgical intervention for BOAS.

**Abbreviations**
BCS: body condition score
BD: bulldog
BOAS: brachycephalic obstructive airway syndrome
FBD: French bulldog
LATE: Laser‐assisted turinoplasty and excision
QVSH: Queens Veterinary School Hospital
RFA: radiofrequency ablation
RFG: respiratory functional grade
WBBP: whole‐body barometric plethysmography

## 1 INTRODUCTION

Brachycephalic obstructive airway syndrome (BOAS) is a common condition in extreme brachycephalic dogs, best characterized in pugs, French bulldogs, and bulldogs. The preferred treatment for most clinically affected dogs is surgical intervention to relieve upper airway obstruction. Although BOAS surgery is not standardized, several techniques have been described, with the choice of procedure depending on individual lesions and the surgeon's preference. Typical interventions include widening the nares, shortening or thinning the soft palate, performing a tonsillectomy, and evaluating and addressing laryngeal collapse.^1^ Immediate postoperative outcomes are well documented, with reported postoperative complications of 6% to 26% and mortality rates of 0.9% to 7%.^2^, ^3^, ^4^, ^5^, ^6^, ^7^, ^8^, ^9^

Brachycephalic obstructive airway syndrome (BOAS) can be evaluated using an exercise stress test to determine a respiratory functional grade (RFG), whole‐body barometric plethysmography (WBBP) to calculate a BOAS index, or owner questionnaires assessing clinical history.¹⁰ These methods have been validated for assessing airway obstruction severity and for distinguishing clinically affected from unaffected dogs.^8^, ^11^, ^12^, ^13^, ^14^

Respiratory functional grading assigns a numerical grade from 0 to 3: grade 0, BOAS free; grade 1, mild BOAS (mild respiratory noise); grade 2, moderate BOAS (requiring medical attention such as weight management, surgical intervention, or both); and grade 3, severe BOAS (requiring immediate surgical intervention). Grades 0 and 1 indicate clinically unaffected dogs, whereas grades 2 and 3 indicate clinical disease.^14^

Whole‐body barometric plethysmography provides a numerical value between 0% and 100%, representing disease severity: 0% indicates no airway obstruction, and 100% indicates the most severe obstruction. Breed‐specific diagnostic cutoffs have been established at a BOAS index greater than 55% for pugs, greater than 49% for French bulldogs, and greater than 44% for bulldogs.^8^, ^14^

Brachycephalic obstructive airway syndrome has been diagnosed and characterized using BOAS indices, which have also been employed to assess the short‐ to medium‐term outcomes of BOAS surgery.^8^ The BOAS indices in dogs improved following traditional multilevel or modified multilevel surgery. After surgery, however, 68% of dogs remained classed as clinically affected by BOAS.^8^

Long‐term outcome studies of BOAS surgery, assessing success in relieving respiratory signs, are based on owner reports and perceptions of improvement. In these studies, the “long term” is defined variably either with reported minimum postoperative follow up of 6–12 months^15^, ^16^ or with reported ranges of follow up of 6–91 months postoperatively.^3^, ^10^, ^17^, ^18^ These studies show good owner satisfaction from surgery and owners report improvement in their dogs’ clinical signs. However, it has also been shown that owners are poor at recognizing signs of BOAS and thus may not be able to assess the surgical outcome accurately.^19^ Objective short‐to‐medium‐term (6 month) assessment in‐person veterinary assessments of postoperative BOAS patients have shown improvement in WBBP BOAS indices compared with preoperative indices.^8^

There are no longer term (>6 months after surgery) studies in outcomes after BOAS surgery using in‐person assessments by a veterinary professional. As BOAS presents early in a dog's life, it is important that studies report the expected outcomes throughout the dog's life, rather than simply the first 6 months after surgery. There are anecdotal reports of outcomes worsening as dogs age after surgery and there is a widely held assumption that BOAS is a progressive condition that deteriorates throughout a dog's life,^20^, ^21^ although this is not supported by definitive evidence. It would thus be reasonable to theorize that results from BOAS surgery may deteriorate over time, particularly as dogs age and muscle strength decreases. Accurate long‐term results are important to aid owners when making decisions about surgical treatment.

The aim of this study was to report and compare objective measurements of preoperative, short‐term postoperative, and long‐term postoperative respiratory outcomes following multilevel BOAS surgery. The study also sought to provide veterinary professionals and owners with an evidence‐based long‐term prognosis and to determine whether improvements in respiratory function after surgery are likely to be maintained. It was hypothesized that the improvements observed at short‐term postoperative assessments would deteriorate in the long term.

## 2 MATERIAL AND METHODS

The study was approved by the ethics and welfare committee of the Department of Veterinary Medicine, University of Cambridge, Cambridge, UK (approval number CR617).

The study included pugs, French bulldogs, and bulldogs referred to the Queens Veterinary School Hospital (QVSH), University of Cambridge, Cambridge, UK that had undergone BOAS surgery before 2019, ensuring a minimum long‐term follow up of 3 years. Exclusion criteria included previous airway surgery prior to referral or any subsequent airway surgery or procedure following the original QVSH surgery. Owners were contacted and dogs were recruited for long‐term assessment.

All dogs underwent preoperative and short‐term postoperative BOAS assessment consultations, which included laryngeal auscultation before and after a 3 min exercise stress test to determine an RFG (Supporting Information, Data S1).^8^, ^12^, ^13^, ^14^, ^18^ and/or BOAS indices generated by WBBP as previously described by Liu et al.^14^ Short‐term postoperative assessments were defined as the first postoperative respiratory assessment after surgery. These were routinely scheduled 8 weeks after surgery but, due to the retrospective nature of these assessments, they were not standardized. If more than one postoperative assessment was made, the first assessment after surgery was used. All short‐term assessments were within 9 months of surgery. Preoperative and short‐term postoperative assessments were performed by a member of the soft tissue surgery team at the QVSH. Long‐term postoperative assessments were performed by the authors (DJ and JL).

### 2.1 Surgical procedure

Multilevel BOAS surgery was defined as two or more of the following procedures to alleviate components of BOAS, chosen according to the individual dog's presentation: stenotic nares (wedge resection of nares^22^ or ala‐vestibuloplasty, including Trader's alaplasty and nasal vestibuloplasty);^8^ overlong or thick soft palate (folded flap palatoplasty^3^ or conventional cut‐and‐sew staphylectomy);^22^ everted tonsils (partial or full tonsillectomy);^8^ laryngeal collapse (sacculectomy^22^ and/or cuneiformectomy). Dogs that had a temporary, prophylactic, or emergency tracheostomy tube removed before hospital discharge were also included. Dogs were excluded if they underwent additional (tertiary referral level) airway procedures such as radiofrequency ablation (RFA) of the tongue, palate, or nasal turbinates, laser‐assisted turbinectomy (LATE),^23^ arytenoid lateralization, or permanent tracheostomy at the time of their primary surgery.

### 2.2 Data collection and means of follow up

Each owner of a dog that met the inclusion criteria was contacted at least twice by phone and/or email. Dogs that met the inclusion criteria were involved in the study. Written client consent for RFG and WBBP was obtained before any assessment was carried out (see the Supporting Information, Data S2). Brachycephalic obstructive airway syndrome assessments included weight measurement, a body condition score (BCS),^24^, ^25^ RFG,^13^ and a 20 min WBBP test.^12^, ^13^, ^14^

Owners completed a questionnaire (in the Supporting Information, Data S3) detailing their dogs’ respiratory and health condition, their experiences of BOAS surgery and their views on their dogs’ current BOAS signs.

### 2.3 Statistical methods and data analysis

A power analysis was conducted using G*Power (Henrich‐Heine University, Düsseldorf, Germany) for sample size estimation to test the study hypothesis. The analysis indicated that 14 subjects were needed to achieve 80% power to detect a large effect (a change of one RFG) and to determine whether long‐term postoperative RFG differed from short‐term postoperative RFG at a significance level of *α* = .05.

All statistical analyses were performed using IBM SPSS Statistics software, version 30.0 (IBM Corp., Armonk, New York, USA). Continuous variables (preoperative, short‐term postoperative, and long‐term postoperative BOAS indices) were assessed for normality using frequency histograms and the Kolmogorov–Smirnov test. Results are reported as means ± standard deviations for normally distributed data and as medians (ranges) for non‐normally distributed data and timings of postoperative assessments. Ordinal variables (RFG) are reported as frequencies and percentages. The BCS has been reported as a binary variable with nonobese (BCS 1–6) and obese (BCS 7–9) reported as frequencies and percentages.

Friedman's test was used to evaluate differences in BOAS indices and RFG across preoperative, short‐term postoperative, and long‐term postoperative assessments. Post hoc analysis was then performed and Wilcoxon signed‐rank tests were conducted, with Bonferroni corrections applied to account for multiple comparisons. An increase in RFG was interpreted as worsening of respiratory function, whereas a decrease was considered an improvement; unchanged RFG values were classified as static. Changes in BOAS index within ±10% were considered equivalent, reflecting the reported interobserver variability in WBBP.^26^ Changes exceeding 10% were classified as improvements (decreases greater than 10%) or deteriorations (increases greater than 10%) in respiratory function. The significance level for all tests was set at .05, unless otherwise specified.

## 3 RESULTS

A total of 153 dogs that underwent BOAS surgery at the QVSH before 2019 met the inclusion criteria and were contacted (see Supporting Information). Of these, 117 (76.5%) responded (Appendix A).

Of owners who were contacted, 31 of 117 (26.5%) declined the invitation to attend a repeat BOAS assessment. Reasons for not attending were given by 15 of 31 (48.4%) (1 of 15 as the dog became dyspneic when traveling; one of 15 as the dog did not tolerate travel for behavioral reasons; two of 15 did not want to participate due to their dogs' neurological issues, and 11 of 15 could not make the available sessions due to distance or personal commitments).

At the time of contact, 21 of 117 (17.9%) of dogs were deceased. Reason for death was provided for eight of 21 dogs (one of eight due to respiratory complications following GA, four of eight due to neurological disease, two of eight neoplasia, one of eight due to cardiac disease). The time of death was provided for 15 of 21 dogs. The median age at time of death was 7.7 years (5–10 years). The median time of death from surgery was 4.5 years (3–6 years).

Thirty‐three of 117 dogs (28.2%) were excluded from further follow up due to undergoing a second upper airway surgical procedure after their original BOAS surgery. No bulldogs underwent a second procedure.

Long‐term postoperative assessments were performed in 32 of 117 (27.3%) dogs. No owner indicated any concurrent cardiac or respiratory disease at the time of long‐term assessment. The median interval to long‐term assessment was 1645 days (range 1208–2927) after surgery. Weight, BCS and long‐term RFG were obtained for all 32 dogs, and 16 of 32 (50%) had BOAS indices available for all three assessments. Table 1 summarizes the preoperative, short‐term and long‐term postoperative assessments.

**TABLE 1:** Preoperative, short‐term, and long‐term postoperative assessment of long‐term assessed dogs.

	Breed (*n* = 32)	Sex (*n* = 32)	Age at surgery (months)	Days since surgery median (range)	BCS (/9)	RFG (*n* = 32)	BOAS index (%) (*n* = 16)
Preoperative assessment	FBD 16 (50%) Pug 10 (31%) BD 6 (19%)	Male 17 (53%) Female 15 (47%)	35 ± 25	N/A	1–6: 25 (78%) 7–9: 7 (22%)	RFG0 0 (0%) RFG1 3 (9%) RFG2 19 (59%) RFG3 10 (31%)	71.6 ± 12.9
Short‐term postoperative assessment				62 (31–237)	1–6: 29 (91%) 7–9: 3 (9%)	RFG0 5 (16%) RFG1 21 (66%) RFG2 5 (16%) RFG3 1 (3%)	46.7 ± 14.4
Long‐term postoperative assessment				1645 (1208–2927)	1–6: 22 (69%) 7–9: 10 (31%)	RFG0 6 (19%) RFG1 18 (56%) RFG2 8 (25%) RFG3 0 (0%)	49.1 ± 18.1

Abbreviations: BCS, body condition score; RFG, respiratory functional grade; BOAS, brachycephalic obstructive airway syndrome; FBD, French bulldog; BD, bulldog; RFG0, grade 0 (BOAS free) on respiratory functional grading; RFG1, grade 1 (mild BOAS) on respiratory functional grading; RFG2, grade 2 (moderate BOAS) on respiratory functional grading; RFG3, grade 3 (severe BOAS) on respiratory functional grading.

The most common combination of surgical procedures was a folded flap palatoplasty, tonsillectomy, sacculectomy, and ala‐vestibuloplasty which was performed in 10 of 32 (31.3%) of dogs. Table 2 shows the surgical procedures used for the dogs that had long‐term assessments.

**TABLE 2:** Surgical procedures applied to dogs who had long‐term postoperative assessments.

Surgical procedures	Number of dogs (*n* = 32)
Folded flap palatoplasty, tonsillectomy, sacculectomy, and alavestibuloplasty	10 (31.3%)
Folded flap palatoplasty, sacculectomy, and ala‐vestibuloplasty	4 (12.5%)
Folded flap palatoplasty, tonsillectomy, sacculectomy, ala‐vestibuloplasty, and bilateral cuneiformectomy	4 (12.5%)
Folded flap palatoplasty, tonsillectomy, ala‐vestibuloplasty	3 (9.4%)
Folded flap palatoplasty and ala‐vestibuloplasty	3 (9.4%)
Folded flap palatoplasty, tonsillectomy, sacculectomy, unilateral cuneiformectomy, and ala‐vestibuloplasty	3 (9.4%)
Folded flap palatoplasty, tonsillectomy, sacculectomy, bilateral cuneiformectomy, ala‐vestibuloplasty, and temporary tracheostomy	2 (6.3%)
Conventional staphylectomy, tonsillectomy, sacculectomy, and ala‐vestibuloplasty	1 (3.1%)
Folded flap palatoplasty and wedge resection of the nostrils	1 (3.1%)
Conventional staphylectomy, tonsillectomy, sacculectomy, unilateral cuneiformectomy, and ala‐vestibuloplasty	1 (3.1%)

### 3.1 Long‐term Respiratory Functional Grades

Preoperative RFGs were as follows: grade 0 (BOAS free), 0 of 32 dogs (0%); grade 1 (mild BOAS), three of 32 (9%); grade 2 (moderate BOAS), 19 of 32 (59%); and grade 3 (severe BOAS), 10 of 32 (31%). Twenty‐five of 32 dogs (78%) had a nonobese BCS, and seven of 32 (22%) had an obese BCS.

Short‐term postoperative RFGs were: grade 0, five of 32 dogs (16%); grade 1, 21 of 32 (66%); grade 2, five of 32 (16%); and grade 3, one of 32 (3%). Twenty‐nine of 32 dogs (91%) had a nonobese BCS, and three of 32 (9%) had an obese BCS.

**FIGURE 1:**
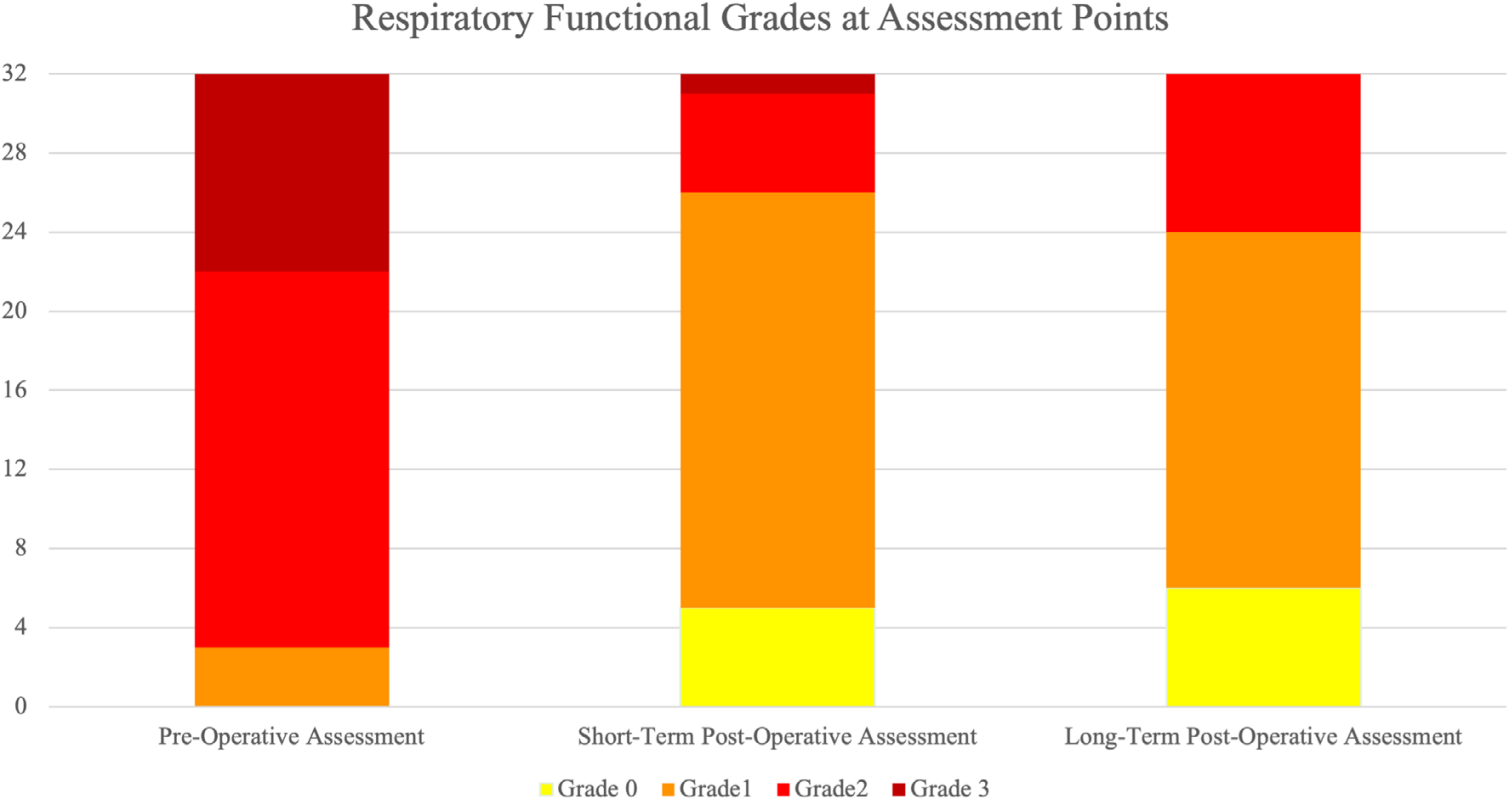
Stacked bar graph showing respiratory functional grades (*n* = 32) at preoperative, short‐term postoperative, and long‐term postoperative assessments.

Long‐term postoperative RFGs were: grade 0, six of 32 dogs (19%); grade 1, 18 of 32 (56%); grade 2, eight of 32 (25%); and grade 3, 0 of 32 (0%). Twenty‐two of 32 dogs (69%) had a nonobese BCS, and 10 of 32 (31%) had an obese BCS (Table 1, Figure 1).

Short‐term and long‐term postoperative assessment RFGs were both improved compared to preoperative assessment RFGs in 26 of 32 (81.3%) dogs and were unchanged compared to preoperative assessment RFGs in six of 32 (18.8%) dogs. In comparison with preoperative assessments, both the short‐term and long‐term assessments found 15 of 32 (46.9%) dogs improved by 1 RFG and 11 of 32 (34.4%) dogs improved by 2 RFGs.

When comparing long‐term to short‐term postoperative RFGs, 20 of 32 (55.6%) dogs maintained the same RFG. Six of 32 (18.8%) had a decrease in one grade and six of 32 (18.8%) had an increase of one grade. Four of six (66%) dogs with an increase in RFG also had an increase in BCS.

Friedman's test showed that there was a difference in RFGs at different assessment time points (*χ* ^2^(2) = 44.087, *p* < .001). Both short‐term postoperative and long‐term postoperative RFGs improved from preoperative RFGs (adjusted *p* < .001, *Z* = −4.604, and *p* < .001, *Z* = −4.604, respectively). Short‐term and long‐term postoperative grades did not differ from each other (adjusted *p* > .999, *Z* = 0.000).

### 3.2 Long‐Term BOAS Indices

**FIGURE 2:**
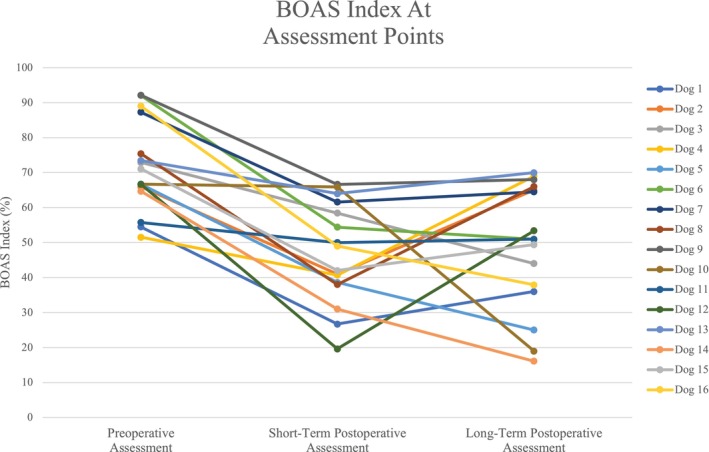
Graph of brachycephalic obstructive airway syndrome (BOAS) index at preoperative, short‐term postoperative, and long‐term postoperative assessments.

Brachycephalic obstructive airway syndrome indices were available for all three time points in 16 dogs: eight of 16 (50%) French bulldogs; five of 16 (31.3%) pugs, and three of 16 (18.8%) bulldogs. Eight of 16 (50%) dogs were male and eight of 16 (50%) were female. The mean preoperative BOAS index was 71.6 ± 12.9%. Thirteen of 16 (87.5%) dogs were nonobese and three of 16 (12.5%) were obese according to their BCS. Short‐term postoperative assessments were a median of 60 days postsurgery, with a range of 31–219 days; the mean BOAS index was 46.7 ± 14.4%. Fourteen of 16 (81.3%) dogs were nonobese and two of 16 (18.8%) were obese according to their BCS score. Long‐term postoperative assessments were a median of 1963 days after surgery, with a range of 1295–2927 days; the mean BOAS index was 49.1 ± 18.1%. Eleven of 16 (68.8%) were nonobese and five of 16 (31.3%) were obese according to their BCS score (Figure 2).

Friedman's test showed there was a difference in BOAS index at different assessment time points (*χ* ^2^(2) = 21.875, *p* < .001). Both short‐term and long‐term postoperative BOAS indices showed improvement compared with preoperative values. From preoperative to short‐term postoperative assessment, the median decrease (improvement) was 25.6% (*p* < .001, *Z* = −3.516). From preoperative to long‐term postoperative assessment, the median decrease was 22.9% (*p* = .003, *Z* = −3.206). Short‐term and long‐term postoperative BOAS indices did not differ (median difference 3.6%, *p* = .623, *Z* = −0.517).

Short‐term postoperative BOAS indices were improved in comparison with preoperative BOAS indices in 13 of 16 dogs (81.3%) or the same in three of 16 (18.8%). No short‐term postoperative BOAS index was worse than at the preoperative assessment. In comparison with preoperative assessments, long‐term postoperative BOAS indices improved in 11 of 16 (68.8%) dogs; they remained constant in four of 16 (25%) dogs, and worsened in one of 16 dogs (6.3%) (Figure 2).

The BOAS indices did not differ between the short‐term and long‐term assessments in seven of 16 (43.8%) dogs. They decreased for two of 16 (12.5%) dogs and increased for four of 16 (25%).

### 3.3 Questionnaire results

Thirty‐one of 32 owners participated in the questionnaire process (97%). Twenty‐nine of 31 (94%) responded that surgery had improved their dogs’ quality of life. Thirty of 31 (97%) of owners would recommend surgery to other owners of dogs with BOAS.

When questioned about whether they considered that their dogs still had breathing problems, 17 of 31 (54.8%) owners felt that their dogs no longer had breathing issues; two of 31 (6.5%) were not sure, and 12 of 31 (38.7%) believed their dog still had breathing problems.

Respiratory functional grades and mean BOAS indices for owners who believed their dogs no longer suffered from breathing problems were as follows: grade 0 (BOAS free), four of 17 (23.5%); grade 1 (mild BOAS), 12 of 17 (63.2%); grade 2 (moderate BOAS), one of 17 (5.9%); and grade 3 (severe BOAS), 0 of 17 (0%). The corresponding mean BOAS index was 50.8 ± 17.6% (*n* = 12). For owners who believed their dogs still suffered from breathing problems, RFGs were: grade 0, two of 12 (16.7%); grade 1, five of 12 (41.7%); grade 2, five of 12 (41.7%); and grade 3, 0 of 12 (0%), with a mean BOAS index of 48.9 ± 20.5% (*n* = 6).

## 4 DISCUSSION

This study presents the first objective, clinician‐assessed long‐term evaluation of respiratory function following BOAS surgery, using both the BOAS index and RFG grading system. Among dogs with long‐term follow up, surgical improvements observed in the short term were maintained without evidence of functional decline. These findings suggest that, in appropriately selected cases, the physiological benefits of BOAS surgery are durable, with short‐term postoperative improvements serving as a reliable indicator of longer term outcomes.

Previous research has focused largely on immediate or short‐term surgical outcomes but long‐term respiratory function after BOAS surgery has remained poorly documented, particularly with objective, standardized assessment tools.^2^, ^3^, ^4^, ^5^, ^6^, ^7^, ^8^, ^9^, ^10^, ^15^, ^16^, ^17^, ^18^ This study helps to address that gap, demonstrating that the combination of multilevel upper airway surgery and structured postoperative monitoring can yield stable functional outcomes over time. Despite the chronic and potentially progressive nature of BOAS, dogs in this cohort did not exhibit worsening of airway function on follow up, even as they aged. This finding is particularly relevant given previous reports that advancing age is associated with increased respiratory signs in brachycephalic dogs. For example, performance on functional tests such as the 1000 m walk has been shown to decline with age in pugs.^20^, ^21^ However, these tests are often confounded by nonrespiratory comorbidity such as orthopedic or neurological disease. In contrast, RFG focuses on respiratory sound patterns during controlled exercise and is less likely to be influenced by such variables. This may explain the apparent stability in functional grade despite increases in patient age and BCS over time.

Although overall respiratory function remained stable, a substantial proportion of dogs continued to show objective evidence of airway obstruction. At long‐term follow up, 25% of dogs had an RFG score of 2, and 59% had a BOAS index exceeding 51%, indicating that clinical signs persisted in many cases. These results underscore the complexity of BOAS, in which anatomical correction alone may not fully eliminate functional compromise. Moreover, they highlight the need for clear definitions of surgical “success,” which may vary depending on whether assessed by objective function or owner perception. A key finding of this study is the observed disconnect between owner‐reported outcomes and objective respiratory data. Several owners reported complete resolution of clinical signs in dogs who remained classified as functionally affected by BOAS. Conversely, some owners expressed concern over dogs with objectively normal respiratory assessments. These results are consistent with previous literature suggesting that brachycephalic dog owners often underestimate the severity of their pets’ respiratory compromise due to the normalization of abnormal signs.^19^, ^21^, ^27^ Subjective improvement is an important outcome of any surgical procedure but these findings reinforce the importance of objective assessment for accurate evaluation and audit.

Surgical procedures in this study were not standardized. All dogs underwent multilevel upper airway surgery but the choice of techniques, including palatoplasty, sacculectomy, rhinoplasty, tonsillectomy, and cuneiformectomy, was tailored to individual anatomical findings. As such, the study reflects real‐world clinical practice but precludes comparison of outcomes by specific technique or breed. Cases undergoing advanced interventions such as laser‐assisted turbinectomy (LATE) or radiofrequency ablation (RFA) were also excluded due to equipment limitations and the tertiary nature of those procedures. Dogs that received revision surgeries were also excluded from the core analysis to preserve the interpretability of time‐matched comparisons. Although this exclusion was methodologically necessary, it introduces potential selection bias by removing dogs with poorer outcomes. Appendix A provides details of these excluded cases, confirming that the long‐term cohort remains broadly representative of the general surgical population at this center.

Although the long‐term cohort was relatively small (32 of 153 dogs), the consistent trends observed in RFG and the BOAS index lend confidence to the conclusion that surgical outcomes were generally maintained. Dogs lost to follow up represented a mix of outcomes and reasons, including geographic distance, unrelated health issues, and in one case, airway disease that limited travel, which further illustrates the representativeness of the evaluated cohort. Future studies may consider travel subsidies or home‐based assessments to improve retention, particularly for more severely affected dogs.

Finally, owner‐based evaluations of BOAS outcome, although widely used, may not be robust enough for surgical audit without validation against objective measures. The future development of postoperative questionnaires that correlate more closely with functional tests like RFG and WBBP would be of considerable value, particularly for general practices without access to specialized diagnostic tools.

In conclusion, the results of this study suggest that short‐term functional improvements following BOAS surgery were sustained in the long term, at least within the subset of dogs with structured follow up.

## CONFLICT OF INTEREST

The authors declare no conflicts of interest related to this report.

## Supporting information


**Data S1.** xxx

**Data S2.** xxx

**Data S3.** xxx

## APPENDIX A PREOPERATIVE AND SHORT‐TERM POSTOPERATIVE ASSESSMENT RESULTS

**Table:**

					Age at Surgery (months)	Preoperative assessment				Short‐term postoperative assessment				
	Breed		Sex			BCS (/9)	RFG		BOAS index (%)	Days since surgery	BCS (/9)	RFG		BOAS index (%)
All dogs (*n* = 153)	FBD	90 (59%)	Male	86 (56%)	30 ± 22	1–6120/153 (78%) 7–9 33/153 (22%)	RFG0	0/153 (0%)	73 ± 18.6 (*n* = 113)	55 (13–286)	1–6125/152 (82%)	RFG0	19/152 (13%)	50.8 ± 18.4 (*n* = 103)
	Pug	43 (28%)	Female	66 (43%)			RFG1	10/153 (7%)			7–9 27/152 (18%)	RFG1	86/152 (57%)	
	BD	20 (13%)					RFG2	87/153 (57%)				RFG2	41/152 (27%)	
							RFG3	56/153 (37%)				RFG3	5/152 (3%)	
Responded to contact (*n* = 117)	FBD	71 (18%)	Male	69 (59%)	29 ± 22	1–6 93/117 (79%) 7–9 24/117 (21%)	RFG0	0 /117 (0%)	73.8 ± 49 (*n* = 90)	55 (14–268)	1–6 96/116 (83%)	RFG0	14/115 (12%)	52.6 ± 18.6 (*n* = 81)
	Pug	32 (27%)	Female	48 (41%)			RFG1	6/117 (5%)			7–9 20/116 (17%)	RFG1	64/115 (56%)	
	BD	14 (15%)					RFG2	65/117 (56%)				RFG2	34/115 (30%)	
							RFG3	46/117 (39%)				RFG3	3/115 (3%)	
Second airway procedure (*n* = 33)	FBD	22 (67%)	Male	19 (58%)	28 ± 16	1–6 27/33 (82%) 7–9 6/33 (18%)	RFG0	0/33 (0%)	81.8 ± 16.3 (*n* = 25)	49 (26–165)	1–6 27/33 (82%)	RFG0	1/33 (3%)	63.1 ± 16 (*n* = 26)
	Pug	11 (33%)	Female	14 (42%)			RFG1	0/33 (0%)			7–9 6/33 (18%)	RFG1	12/33 (36%)	
	BD	0 (0%)					RFG2	17/33 (52%)				RFG2	18/33 (55%)	
							RFG3	16/33 (48%)				RFG3	2/33 (6%)	
Deceased at time of long‐term assessment (*n* = 21)	FBD	12 (57%)	Male	12 (57%)	36 ± 20	1–6 15/21(71%) 7–9 6/21 (29%)	RFG0	0/21 (0%)	72 ± 27.4 (*n* = 14)	49 (14–90)	1–6 14/20 (70%)	RFG0	1 /19 (5%)	54.8 ± 19.2 (*n* = 12)
	Pug	4 (19%)	Female	9 (43%)			RFG1	1/21 (5%)			7–9 6/20 (30%)	RFG1	10/19 (53%)	
	BD	5 (24%)					RFG2	6/21 (29%)				RFG2	8/19 (42%)	
							RFG3	14/21 (67%)				RFG3	0/19 (0%)	
Declined long‐term assessments (*n* = 31)	FBD	21 (68%)	Male	21 (68%)	27 ± 12	1–6 27/31 (87%) 7–9 4/31 (13%)	RFG0	0/31 (0%)	69 ± 18.6 (*n* = 23)	58 (20–286)	1–6 27/31 (87%)	RFG0	7/31 (23%)	46.1 ± 18.4 (*n* = 12)
	Pug	4 (13%)	Female	10 (32%)			RFG1	2/31 (6%)			7–9 4/31 (13%)	RFG1	21/31 (68%)	
	BD	3 (10%)					RFG2	23/31 (74%)				RFG2	3/31 (10%)	
							RFG3	6/31 (19%)				RFG3	0/31 (0%)	
Long‐term assessed cohort (*n* = 32)	FBD	16 (50%)	Male	17 (44%)	35 ± 25	1–6 25/32 (78%) 7–9 7/32 (22%)	RFG0	0/32 (0%)	71.6 ± 5.2 (*n* = 26)	84 (31–237)	1–6 29/32 (91%)	RFG0	5/32 (16%)	44.7 ± 14.7 (*n* = 28)
	Pug	10 (31%)	Female	15 (47%)			RFG1	3/32 (9%)			7–9 3/32 (9%)	RFG1	21/32 (66%)	
	BD	6 (19%)					RFG2	19/32 (59%)				RFG2	5/32 (16%)	
							RFG3	10/32 (31%)				RFG3	1/32 (3%)	

BCS, body condition score; RFG, respiratory functional grade; BOAS, brachycephalic obstructive airway syndrome; FBD, French bulldog; BD, bulldog; RFG0, grade 0, BOAS free on respiratory functional grading; RFG1, grade 1, mild BOAS on respiratory functional grading; RFG2, grade 2, moderate BOAS on respiratory functional grading; RFG3, grade 3 severe BOAS on respiratory functional grading.

## References

[1] Wallace ML . Surgical management of brachycephalic obstructive airway syndrome: an update on options and outcomes. Vet Surg. 2024;53(7):1173‐1184. 38952039 10.1111/vsu.14131

[2] Seneviratne M , Kaye BM , Ter Haar G . Prognostic indicators of short‐term outcome in dogs undergoing surgery for brachycephalic obstructive airway syndrome. Vet Rec. 2020;187(10):403. 32764033 10.1136/vr.105624

[3] Findji L , Dupré G . Folded flap palatoplasty for treatment of elongated soft palates in 55 dogs. Eur J Companion Anim Pract. 2009;19(2):125‐132.

[4] Ree JJ , Milovancev M , Townsend KL . Factors associated with major complications in the short‐term postoperative period in dogs undergoing surgery for brachycephalic airway syndrome. Can Vet J. 2016;57(9):976‐980. 27587891 PMC4982570

[5] Fasanella FJ , Shivley JM , Wardlaw JL , Givaruangsawat S . Brachycephalic airway obstructive syndrome in dogs: 90 cases (1991–2008). J Am Vet Med Assoc. 2010;237(9):1048‐1051. 21034343 10.2460/javma.237.9.1048

[6] Tarricone J , Hayes GM , Singh A , Davis G . Development and validation of a brachycephalic risk (BRisk) score to predict the risk of complications in dogs presenting for surgical treatment of brachycephalic obstructive airway syndrome. Vet Surg. 2019;48(7):1253‐1261. 31350865 10.1111/vsu.13291

[7] Hughes JR , Kaye BM , Beswick AR , Ter Haar G . Complications following laryngeal sacculectomy in brachycephalic dogs. JSAP. 2018;59(1):16‐21. 10.1111/jsap.12763 29047114

[8] Liu NC , Oechtering GU , Adams VJ , Kalmar L , Sargan DR , Ladlow JF . Outcomes and prognostic factors of surgical treatments for brachycephalic obstructive airway syndrome in 3 breeds. Vet Surg. 2017;46(2):271‐280. 28146288 10.1111/vsu.12608

[9] Filipas MC , Owen L , Adami C . A retrospective observational cohort study on the postoperative respiratory complications and their risk factors in brachycephalic dogs undergoing BOAS surgery: 199 cases (2019‐2021). JSAP. 2024;65(5):329‐337. 10.1111/jsap.13707 38413137

[10] Poncet CM , Dupre GP , Freiche VG , Bouvy BM . Long‐term results of upper respiratory syndrome surgery and gastrointestinal tract medical treatment in 51 brachycephalic dogs. JSAP. 2006;47(3):137‐142. 10.1111/j.1748-5827.2006.00057.x 16512845

[11] Ladlow J . Brachycephalic obstructive airway syndrome: guide to the respiratory functional grading scheme. In Pract. 2021;43(10):548‐555.

[12] Liu NC , Sargan DR , Adams VJ , Ladlow JF . Characterisation of brachycephalic obstructive airway syndrome in French bulldogs using whole‐body barometric plethysmography. PLoS One. 2015;10(6):e0130741. 26079684 10.1371/journal.pone.0130741 PMC4469695

[13] Riggs J , Liu N , Sutton DR , Sargan D , Ladlow JF . Validation of exercise testing and laryngeal auscultation for grading brachycephalic obstructive airway syndrome in pugs, French bulldogs, and English bulldogs by using whole‐body barometric plethysmography. Vet Surg. 2019;48(4):488‐496. 30666670 10.1111/vsu.13159

[14] Liu NC , Adams VJ , Kalmar L , Ladlow JF , Sargan DR . Whole‐body barometric plethysmography characterizes upper airway obstruction in 3 brachycephalic breeds of dogs. J Vet Intern Med. 2016;30(3):853‐865. 27159898 10.1111/jvim.13933 PMC4913582

[15] Pohl S , Roedler FS , Oechtering GU . How does multilevel upper airway surgery influence the lives of dogs with severe brachycephaly? Results of a structured pre‐and postoperative owner questionnaire. Vet J. 2016;210:39‐45. 26897434 10.1016/j.tvjl.2016.01.017

[16] Riecks TW , Birchard SJ , Stephens JA . Surgical correction of brachycephalic syndrome in dogs: 62 cases (1991–2004). J Am Vet Med Assoc. 2007;230(9):1324‐1328. 17472557 10.2460/javma.230.9.1324

[17] Carabalona JPR , Le Boedec K , Poncet CM . Complications, prognostic factors, and long‐term outcomes for dogs with brachycephalic obstructive airway syndrome that underwent H‐pharyngoplasty and ala‐vestibuloplasty: 423 cases (2011‐2017). J Am Vet Med Assoc. 2021;260 (S1):S65‐S73. 34914621 10.2460/javma.20.09.0534

[18] Torrez CV , Hunt GB . Results of surgical correction of abnormalities associated with brachycephalic airway obstruction syndrome in dogs in Australia. JSAP. 2006;47(3):150‐154. 10.1111/j.1748-5827.2006.00059.x 16512847

[19] Packer R , Hendricks A , Burn C . Do dog owners perceive the clinical signs related to conformational inherited disorders as “normal” for the breed? A potential constraint to improving canine welfare. UFAW. 2012;21(S1):81‐93.

[20] Aromaa M , Rajamäki M , Lilja‐Maula L . A follow‐up study of exercise test results and severity of brachycephalic obstructive airway syndrome signs in brachycephalic dogs. UFAW. 2021;30(4):441‐448.

[21] Aromaa M , Lilja‐Maula L , Rajamäki M . Assessment of welfare and brachycephalic obstructive airway syndrome signs in young, breeding age French bulldogs and pugs, using owner questionnaire, physical examination and walk tests. UFAW. 2019;28(3):287‐298.

[22] Monnet E . Brachycephalic airway syndrome. In: Slatter D , ed. Textbook of Small Animal Surgery. 3rd ed. Saunders; 2003:808‐813.

[23] Liu N , Genain M , Kalmar L , Sargan DR , Ladlow JF . Objective effectiveness of and indications for laser‐assisted turbinectomy in brachycephalic obstructive airway syndrome. Vet Surg. 2019;48(1):79‐87. 30303538 10.1111/vsu.13107

[24] ROYAL CANIN SAS . Royal Canin Body Condition Score Charts. 2013 https://www.royalcanin.co.uk/wp-content/uploads/2017/02/BCS-chart-03.12.13.pdf.

[25] Cambridge BOAS . Research group. Body Condition Scoring in Pugs. 2017. https://www.vet.cam.ac.uk/files/media/Pug_health_scheme_BCS_v2.jpg.

[26] Liu NC . Novel Diagnostic Test for Brachycephalic Obstructive Airway Syndrome (BOAS) Using Whole ‐ Body Barometric Plethysmography (WBBP). University of Cambridge; 2016.

[27] Packer RMA , O'Neill DG , Fletcher F , Farnworth MJ . Great expectations, inconvenient truths, and the paradoxes of the dog‐owner relationship for owners of brachycephalic dogs. PLoS One. 2019;14(7):e0219918. 31323057 10.1371/journal.pone.0219918 PMC6641206

