# Supplementary material for: Comparison of short‐ and long‐term objective respiratory outcomes after surgery for brachycephalic obstructive airway syndrome
Source: Vet Surg. 2025 Oct 18;55(1):59–68. doi: 10.1111/vsu.70034 (PMC12810434; doi:10.1111/vsu.70034)
Supplement: Supplementary file 1 — Data S1. xxx [file VSU-55-59-s001.docx]

**French Bulldogs and Bulldogs**
