# Supplementary material for: Comparison of short‐ and long‐term objective respiratory outcomes after surgery for brachycephalic obstructive airway syndrome
Source: Vet Surg. 2025 Oct 18;55(1):59–68. doi: 10.1111/vsu.70034 (PMC12810434; doi:10.1111/vsu.70034)
Supplement: Supplementary file 2 — Data S2. xxx [file VSU-55-59-s003.pdf]

The Queen's Veterinary School Hospital
University of Cambridge
Madingley Road Cambridge CB3 0ES

Telephone: 01223 337621 Fax: 01223 330848

Jane Ladlow MA, VetMB, CertSAS, CertVR, DipECVS, MRCVS
European Specialist in Small Animal Surgery
Soft Tissue Surgeon

Dear ………………,

We are currently investigating long term breathing function after brachycephalic obstructive airway syndrome (BOAS) surgery in pugs, French bulldogs, and bulldogs.

We have contacted you as your dog was treated at Cambridge within the last 8 years.

We would like to assess how your dog progressed after surgery with a questionnaire and also, if possible, by assessing your dog again at the clinic.

It is difficult to fully assess a dog's level of BOAS (airway obstruction) from physical exam alone therefore we request your permission to perform a better assessment of your animal which would include two additional non-invasive tests to assess breathing function: a clinical grading scheme with an exercise tolerance test and a plethysmography chamber. The exercise test is a three-minute trot test, and the plethysmography chamber is a clear plastic box which the animals stand or sit in whilst pressure changes caused by breath being warmed when inspired are measured. These pressure changes are then translated to inspiratory and expiratory function.

These assessments were performed prior to any after your dog's initial surgery and any dog that does not tolerate these assessments of breathing function will be withdrawn.

There will be no charge for these assessments.

You are free to withdraw your dog from the study at anytime. If you have any concerns about the study please contact Jane Ladlow, jfl1001@cam.ac.uk.

Yours sincerely,

Jane Ladlow MA VetMB, CertVR, CertSAS, DipECVS MRCVS

European Specialist in Veterinary Surgery

Email: jfl1001@cam.ac.uk

Daisy Johnson BVetMed PgC(SAS) MRCVS

Junior Clinical Training Scholar.

Email: daj52@cam.ac.uk

I give consent for my dog ……………………to participate in the respiratory function tests described above.

Signature………………………………… Date ……………………………………
