# Supplementary material for: Comparison of short‐ and long‐term objective respiratory outcomes after surgery for brachycephalic obstructive airway syndrome
Source: Vet Surg. 2025 Oct 18;55(1):59–68. doi: 10.1111/vsu.70034 (PMC12810434; doi:10.1111/vsu.70034)
Supplement: Supplementary file 3 — Data S3. xxx [file VSU-55-59-s002.pdf]

Brachycephalic Obstructive Airway Syndrome (BOAS) Research Group

Queen’s Veterinary School Hospital (QVSH)
Department of Veterinary Medicine
University of Cambridge
Madingley Road Cambridge CB3 0ES
QVSH Tel: 01223 337621 Email: hospital@vet.cam.ac.uk
BOAS lab Tel: 01223 337690 Email: vetboas@vet.cam.ac.uk

Brachycephalic Obstructive Airway Syndrome (BOAS) Post Surgery Questionnaire

Introduction:

Thank you for participating in our study. The first section is on your dog’s respiratory health in relation to BOAS. The second section is general health questions. Please fill in blank spaces or tick boxes as required. We very much appreciate your help with our research; your participation is voluntary and can be withdrawn at any time. Please be assured that any identifying information will remain confidential.

Date: ____________________

Owner name: _____________________________

Email address: ____

Contact number: ___________________________

Address (optional): ____

Dog name: _______________________________

Breed: [☐ Pug] [☐ French Bulldog] [☐ English Bulldog]

Kennel Club registered: [☐ No] [☐ Yes (KC registration number if known: ______________)]

Microchip number (if known): _____________________________

Age: _________ year(s) and ____________ month(s)

Gender: [☐ Male] [☐ Female]

Neuter status: [☐ Intact] [☐ Neutered]

Brachycephalic Obstructive Airway Syndrome (BOAS) Research Group

Queen's Veterinary School Hospital (QVSH)
Department of Veterinary Medicine
University of Cambridge
Madingley Road Cambridge CB3 0ES
QVSH Tel: 01223 337621 Email: hospital@vet.cam.ac.uk
BOAS lab Tel: 01223 337690 Email: vetboas@vet.cam.ac.uk

Respiratory Health Section

Q1. How much exercise does your dog get on average in a day?

_________ minutes [☐ Not sure]

Q2a. Does your dog have loud noisy breathing while awake at rest currently?

[☐ Never] [☐ Rarely (weekly/fortnightly)] [☐ Frequently (daily)] [☐ Always] [☐ Not sure]

Q2b. If yes is this better or worse than prior to original surgery?

[☐ Didn't do prior to surgery]

[☐ Improved since surgery]

[☐ Originally improved with surgery but now as bad/worse]

[☐ Surgery did not change this]

[☐ Not sure]

Q3a. Does your dog snore loudly when sleeping?

[☐ Never] [☐ Rarely (weekly/fortnightly)] [☐ Frequently (daily)] [☐ Always] [☐ Not sure]

Q3b. If yes is this better or worse than prior to original surgery?

[☐ Didn't do prior to surgery]

[☐ Improved since surgery]

[☐ Originally improved with surgery but now as bad/worse]

[☐ Surgery did not change this]

[☐ Not sure]

Q4 Does your dog have loud noisy breathing during physical exercise?

[☐ Never] [☐ Rarely (weekly/fortnightly)] [☐ Frequently (daily)] [☐ Always] [☐ Not sure]

Q4a. If yes is this better or worse than prior to original surgery?

[☐ Didn't do prior to surgery]

[☐ Improved since surgery]

[☐ Originally improved with surgery but now as bad/worse]

[☐ Surgery did not change this]

[☐ Not sure]

Brachycephalic Obstructive Airway Syndrome (BOAS) Research Group

Queen’s Veterinary School Hospital (QVSH)
Department of Veterinary Medicine
University of Cambridge
Madingley Road   Cambridge CB3 0ES
QVSH   Tel: 01223 337621   Email: hospital@vet.cam.ac.uk
BOAS lab   Tel: 01223 337690   Email: vetboas@vet.cam.ac.uk

Q5 Does your dog have issues with their breathing during hot weather?

[☐ Never]   [☐ Rarely (weekly/fortnightly)]   [☐ Frequently (daily)]   [☐ Always]   [☐ Not sure]

Q5a. If yes is this better or worse than prior to original surgery?

[☐ Didn’t do prior to surgery/worse than prior to surgery]

[☐ Improved since surgery]

[☐ Originally improved with surgery but now as bad/worse]

[☐ Surgery did not change this]

[☐ Not sure]

Q6. Has your dog ever collapsed because of problems with breathing?

[☐ Never]   [☐ Yes]   [☐ Not sure]

Q6.a. If yes, how many times have you noted this episode?

[☐ Has done pre and post surgery]   [☐ Only did pre-surgery]   [☐ Only has post surgery]   [☐ Not sure]

Q7. Has your dog ever been cyanotic (i.e. blue-purple tongue)?

[☐ Never]   [☐ Yes]   [☐ Not sure]

Q7a. If yes is this better or worse than prior to original surgery?

[☐ Yes Prior to surgery but never since]

[☐ Yes prior to surgery but rarely since]

[☐ Yes same frequency pre and post surgery]

[☐ Yes and worse since surgery]

[☐ Not sure]

Q8. Does your dog ever had reverse sneezing (i.e. backwards sneezing)?

[☐ Never]   [☐ Rarely (weekly/fortnightly)]   [☐ Frequently (daily)]   [☐ Not sure]

Q8a. If yes is this better or worse than prior to original surgery?

[☐ Didn’t do prior to surgery]

[☐ Less frequent since surgery]

[☐ Originally less frequent after surgery but now as bad/worse]

[☐ Surgery did not change this]

[☐ Not sure]

Brachycephalic Obstructive Airway Syndrome (BOAS) Research Group

Queen's Veterinary School Hospital (QVSH)
Department of Veterinary Medicine
University of Cambridge
Madingley Road Cambridge CB3 0ES
QVSH Tel: 01223 337621 Email: hospital@vet.cam.ac.uk
BOAS lab Tel: 01223 337690 Email: vetboas@vet.cam.ac.uk

Q9. Does your dog have any of the following problems with eating and digestion?

		Never	Did prior to original surgery. Does not now	Did prior to surgery and no change since surgery	Reduced frequency since surgery	Worse since surgery	Not sure
**9a.**	Regurgitation (bringing up undigested food/liquid) or vomiting	☐	☐	☐	☐	☐	☐
**9b.**	Choking on food (struggling to swallow, gagging/choking after eating)	☐	☐	☐	☐	☐	☐
**9c.**	Out of breath during eating	☐	☐	☐	☐	☐	☐

Q10. Do you notice any of the following with your dog when sleeping?

		Never	Did prior to original surgery	No change since surgery	Reduced/improved frequency since surgery	Worse since surgery	Not sure
**10a.**	Waking up a few times during sleep	☐	☐	☐	☐	☐	☐
**10b.**	Restlessness at night	☐	☐	☐	☐	☐	☐
**10c.**	Sleeps with chin in elevated position and neck extended	☐	☐	☐	☐	☐	☐
**10d.**	Attempting to sleep in a sitting position	☐	☐	☐	☐	☐	☐
**10e.**	Episodes of sleep apnea (stopping breathing temporarily when asleep, then jolting awake to take a breath)	☐	☐	☐	☐	☐	☐
**10f.**	Sleeping with their eyes open	☐	☐	☐	☐	☐	☐

Q11. Do you think your dog still suffers from breathing problems?

[☐ NO]

[☐ Yes; Currently worse than ever]

[☐ Yes surgery did not change this]

[☐ Yes; Worse than in the first 6-12m post-surgery but better than pre-surgery]

[☐ Yes but stable since initial surgery]

[☐ Not sure]

Q12. Has your dog undergone any further upper airway surgery?

[☐ No] [☐ Yes] [☐ Not sure]

Q12a. If yes, what were the surgical procedures and when/where:

____

____

General Health Section

Q13. Has your dog ever been affected or is currently affected by any other health issues that may affect their breathing such as heart disease or other respiratory disease other than BOAS

[☐ No] [☐ Yes] [☐ Not sure]

Q13a. If yes, please include further details (medication, investigations undertaken):

____

____

____

____

Q12. Has your dog ever been affected or is currently affected by any other health issues other than breathing problems? (For example: skin disease, disc disease, fits/seizures)

[☐ No] [☐ Yes] [☐ Not sure]

Q13a. If yes, please include further details (medication, investigations undertaken):

____

____

____

____

Brachycephalic Obstructive Airway Syndrome (BOAS) Research Group

Queen's Veterinary School Hospital (QVSH)
Department of Veterinary Medicine
University of Cambridge
Madingley Road Cambridge CB3 0ES
QVSH Tel: 01223 337621 Email: hospital@vet.cam.ac.uk
BOAS lab Tel: 01223 337690 Email: vetboas@vet.cam.ac.uk

Q13. Is your dog currently on any medications?

[☐ No] [☐ Yes] [☐ Not sure]

Q12a. If yes, please include further details:

Q14. Do you think airway surgery improved your dog's quality of life?

[☐ No] [☐ Yes] [☐ Not sure]

Q15. Would you recommend surgery to other owners of dogs suffering from BOAS?

[☐ No] [☐ Yes] [☐ Not sure]

Thank you very much for taking part. Occasionally, we may wish to get in touch with you for a follow-up in our research. Would you be happy for us to contact you for the purpose of the research study?

[☐ Yes] [☐ No] **If yes, contact preference:** [☐ Telephone] [☐ Email]
